# Immunohistochemical Study of *Ezrin* Expression in Colorectal Carcinoma: A Comparative Study between Objective Method and Digital Quantitative Assessment

**DOI:** 10.31557/APJCP.2020.21.4.967

**Published:** 2020-04

**Authors:** Essam E Ayad, Yousra O Kamal eldin, Ali A El-hindawi, Mona S Abdelmagid, Hesham A Elmeligy

**Affiliations:** 1 *Department of Pathology, Faculty of Medicine, Cairo University, Cairo, *; 2 *Department of Pathology, Faculty of Medicine, October 6 University, *; 3 *Department of General Surgery, Theodor Bilharz Research Institute, Giza, Egypt. *

**Keywords:** Colorectal carcinoma, ezrin, immunohistochemical expression, objective analysis, quantitative analysis

## Abstract

**Background:**

Colorectal cancer is one of the leading causes of cancer death in both developed and developing nations. It is the third most common type of cancer and the fourth leading cause of cancer-related deaths worldwide. Ezrin is involved in maintaining cell structure and cell motility. Expression levels of the *ezrin* gene correlate with numerous human malignancies.

**Material and Methods:**

Ezrin expression was evaluated in fifty one cases of colorectal carcinoma by using two methods; objective and quantitative method to determine the statistical relation between ezrin objective analysis score and clinicopathological parameters and to do a comparative study between both methods of analysis.

**Results:**

*Ezrin* was expressed in 92.2% of cases, and it showed a statistical significant relation with tumor grade. A statistically significant relation was found between ezrin objective analysis score and ezrin quantitative analysis score (P-value <0.05). A strong positive Pearson correlation exists between both methods of analysis (R=0.868).

**Conclusion:**

Ezrin has a role in colorectal cancer progression and it might provide clinically valuable information in predicting the behavior of colorectal cancer. Digital pathology offers the potential for improvements in quality, efficacy and safety. It will be necessary to carry out similar studies on a larger sample size in order to elucidate the possible prognostic significance of ezrin in colorectal carcinoma and ensure the ability of digital pathology to transform the practice of diagnostic pathology.

## Introduction

Colorectal cancer (CRC) is the third most common malignant neoplasm worldwide after lung and breast cancer and represents the fourth most common cause of cancer related mortality (American Cancer Society, 2015). Tumor metastasis starts with breakdown of epithelial integrity, followed by malignant cells invading into the surrounding stroma and lymphovascular space by which tumor cells travel to distant target organs (Elzagheid et al., 2006). 

Cell adhesion molecules and actin cytoskeleton play a crucial role in tumor metastasis (Hunter, 2004). The primary mechanism for most types of cell migration is the actin cytoskeleton remodeling (Yu et al., 2004). 

The ezrin gene is a member of the ezrin-radixin-moesin (ERM) cytoskeleton-associated protein family and is involved in a wide variety of cellular processes. It is one of the components of cell-surface structures involved in cell adhesion to the extracellular matrix and has been implicated in membrane-cytoskeleton interactions (Fehon et al., 2010; Neisch and Fehon, 2011).

Many publications showed that *ezrin* is strongly expressed in a variety of invasive cancers including osteosarcoma, melanoma, soft tissue sarcoma, pancreatic carcinoma, hepatocellular carcinoma, gastric and breast carcinoma (Makitie et al., 2001; Weng et al., 2005; Meng et al., 2010; Wu et al., 2011; Chen et al., 2011; Zheng et al., 2011; Fan et al., 2011; Zhu et al., 2012).

The ezrin protein correlates with tumor invasiveness, metastasis and clinical prognosis in numerous types of human cancer including colorectal carcinoma (Xie et al., 2011; Korkeila et al., 2011). 

Although advances have been made while studying the molecular basis of this disease, the spectrum of genes that reveal altered expression in colorectal carcinoma as well as their role in the disease remain unclear (Carlisle et al., 2012). Therefore, more sensitive colorectal carcinoma biomarkers that are capable of predicting prognosis and guiding effective targeting therapy are required.

The molecular characteristics of the ezrin protein may be important during tumor progression (Schlecht et al., 2012); however, the clinical significance of these characteristics in human cancer requires clarification.

Worldwide, digital pathology and whole slide imaging is being used increasingly in research applications, frozen section and consultation. It has the potential to transform the practice of diagnostic pathology (Griffin and Treanor, 2017).

Our study differs from other studies that was done for evaluating ezrin immunohistochemical stain in colorectal cancer in that we did complete digitalization of the immunostained slides and analyzed *ezrin* expression by quantitative method. We also added a comparative study between *ezrin* expression by objective method of analysis (pathologist visual score) and *ezrin* expression by quantitative method of analysis (digital score). So, we have succeeded in the pathology department, Kasr Al-Ainy hospital for the first time to do digital quantitation for a marker with cytoplasmic expression.

The aim of the study is to determine the significance of ezrin in the development and progression of colorectal carcinoma by evaluating its expression and determining its relation with clinicopathological parameters including gender, age, tumor histopathological type, site, gross appearance, size, histological grade, tumor stage and lymph node status to provide the evidence for clinical prognosis. We also aimed at doing a comparative study between *ezrin* expression by objective method and *ezrin *expression by quantitative method to ensure the value of digital pathology by assessing the utility of quantitative digital analysis of the IHC stained slides.

## Materials and Methods

*Patient’s cohort*

We analyzed 51 cases of colorectal carcinoma collected for the study from the Pathology Department, Faculty of Medicine, Cairo University during the period between 2014 and 2016. Histological sections were obtained from the paraffin blocks of 51 total colectomy specimens.

*Immunohistochemistry for ezrin*

We obtained 4 microns thick from each paraffin block which contained formalin fixed tumor tissue. During the whole staining procedure the slides were treated with an autostainer (Dako autostainer link 48) using a polymer-based detection system (Dako En Vision TM FLEX, K8000) using the anti-ezrin antibody ( by monoclonal mouse anti-ezrin) obtained from Thermo Fisher Scientific (UK) and used at a dilution of 1:50. Then all the stained slides were scanned by Biolmagene slide scanner [in the Digital Pathology Unit, Pathology Department, Faculty of Medicine, Cairo University]. A section of renal cell carcinoma, which is mentioned to be a positive control for ezrin in the marker datasheet, was used as a positive external control in the current study and the adjacent normal mucosa was used as an internal positive control.

*Evaluatin of ezrin expression*

*Was done by using two methods:*

1) Objective method: using light microscopy, brown membranous or cytoplasmic staining was accepted as ezrin immunoreactivity. The assessment of *ezrin* expression was evaluated by analysis of the staining intensity and the percentage of the stained cells. Staining intensity was scored as 0 (negative), 1 (weak), 2 (moderate) or 3 (strong). The percentage of stained cells was categorized as 0 (no staining), 1 (1-25 %), 2 (26 -50 %), 3 (51-75 %) and 4 (76 -100 %). The overall score was expressed as the sum of the intensity and the percentage scores with the product ranging from 0-7. On the bases of the final score, expression was categorized as negative (0-1), weakly positive (2-4) and strongly positive (5-7) (Lin and Chen, 2013).

2) Quantitative method: scanning of the glass slides was done using the Biolmagene slide scanner [in the Digital Pathology Unit, Pathology Department, Faculty of Medicine, Cairo University] at x20.

*The virtual slides obtained were JP2 format*

Viewing the slides and selecting the snapshots were done using the Biolmagene’s Image Viewer, Version: 2.0.0.1-RC2, 2005.

Twenty snapshots were selected from each virtual slides of the 51 cases included in the study [The immunohistochemical positively charged slides].

All the snapshots underwent the Digital Quantitative analysis of the ezrin immunostain positivity using QuPath Software, Version: 0.1.2, 2016 for detection and quantitative analysis of the Cytoplasmic Immuno-histochemical stains.

We took the average of the percentage of quantitative analysis of the snapshots of each virtual slides and assessed *ezrin* expression using the above mentioned method.

*Statistical analysis*

Microsoft excel 2013 was used for data entry and the statistical package for social science (SPSS) version 21 (SPSS, Armonk, New York: International Business Machines Corporation) was used for data analysis. 

Simple descriptive statistics (arithmetic mean and standard deviation) used for summary of quantitative data and frequencies used for qualitative data.

Bivariate relationship was displayed in cross tabulations and Comparison of proportions was performed using the chi-square test. 

One-way Annova and post-hook tests were used to compare normally distributed quantitative data.

Pearson correlation (R) value was used to compare normally distributed quantitative data. {R explanation: positive or negative according to the sign, <0.5 weak correlation, between 0.5 - 0.7 moderate and >0.7 strong}.

The level of significance was set at probability (P) value <0.05.

## Results

*Clinicopathological results*

This study included 51 cases of colorectal carcinoma that were classified into 45 cases of conventional adenocarcinoma, 3 mucinous, 1 neuroendocrine and 2 papillary adenocarcinoma, there were slight female predominance, 51% of the studied cases were females and 49% were males, with female to male : ratio 1.04 : 1. The mean age of the studied patients with colorectal carcinoma was 57 years, ranging from 21 to 83 years old. About 90.2% of the patients were older than 40 years while 9.8% of the patients were younger than or equal to 40 years. In our study the most frequent site of tumor involvement was the right side and represented 41.2% of cases. With respect to gross appearance of the tumor, 41.2% of colorectal carcinomas were ulcerating, 39.2% fungating, while 19.6 % were infiltrating. The mean size of the tumor in the studied cases was 5.5 cm. According to the grade of differentiation there were no well differentiated cases, 86.3% were moderately differentiated and 13.7% were poorly differentiated. Regarding tumor invasion (T) in this study, most of the studied cases were T3 representing 72.5%. Concerning lymph node status (N) in this study, 51% of cases showed no lymph node metastasis (N0). In our study nearly all cases (98%) showed no distant metastasis (M0) (Table 1).

*Ezrin immunohistochemical (IHC) results*

*Ezrin IHC* expression was focally positive in the adjacent normal mucosa, but its expression was significantly higher in colorectal carcinoma tissue. *Ezrin IHC* expression was strongly positive in 56.9% of the studied cases (29 out of 51 cases), weakly positive in 35.3% of the studied cases (18 out of 51 cases) and negative in 7.8% of them (4 out of 51 cases) (Figure 3, 4, 5 and 6). There was a statistically significant relation between *ezrin IHC* expression and tumor grade (*P*- value = 0.046).

*Ezrin immunohistochemical (IHC) results by objective analysis versus quantitative analysis*

**Figure 1:**
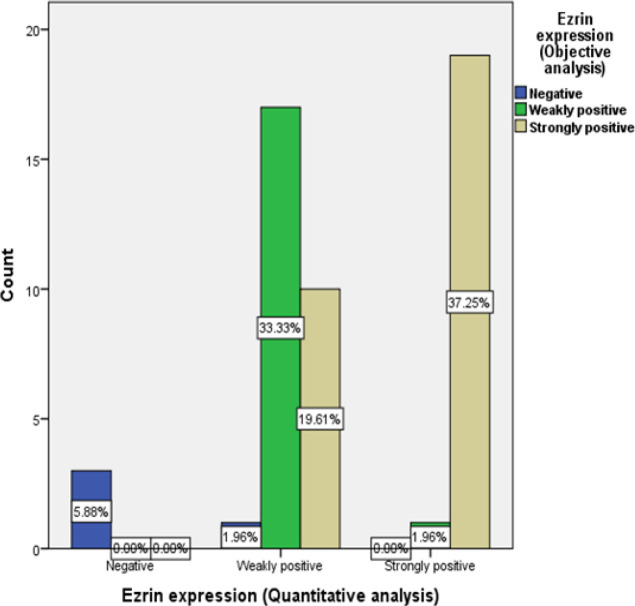
Illustrating the Relation between Ezrin IHC Expression Using Objective Analysis and Quantitative Analysis

**Figure 2:**
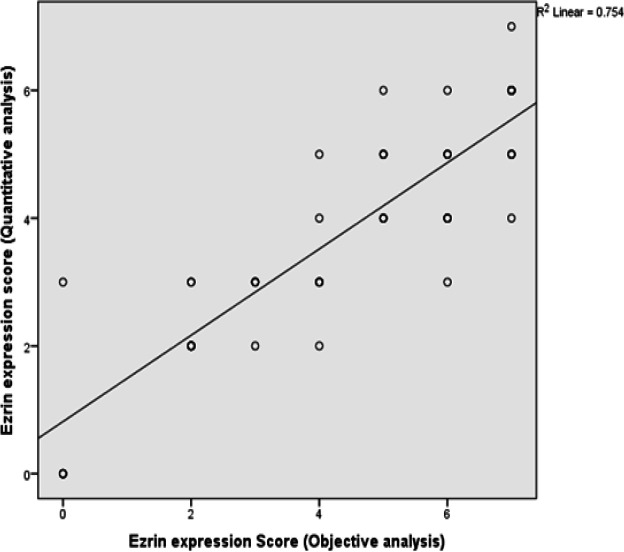
Illustrating the Correlation between Ezrin IHC Expression Objective Analysis and Ezrin IHC Expression Quantitative Analysis

*Ezrin* expression was strongly positive in 39.2% (20 out of 51 cases), weakly positive in 54.9% (28 out of 51 cases) and negative in 5.9% of the studied cases (3 out of 51 cases) analyzed by the quantitative method of analysis. Statistically significant relation was found between ezrin IHC results by objective analysis and by quantitative analysis regarding negative and weakly positive cases (P-value = <0.001), negative and strongly positive cases (P-value = <0.001) and weakly positive and strongly positive cases (P-value = <0.001). Statistically signifiant relation was found between both methods of analysis (p-value<0.005) (Table 2 and Figure 1). A strong positive correlation existed between both expressions (P value <0.001) (R value= 0.868) (Figure 2).

## Discussion

*Ezrin* expression is higher in colorectal cancer tissues than in adjacent normal mucosa and the high level of *ezrin* expression is closely related to the colorectal cancer invasion and metastatic process (Wang et al., 2009). 

Elzagheid et al., (2008) suggest that ezrin may play a role in colorectal cancer progression and that *ezrin *expression might provide clinically valuable information in predicting the biological behavior of colorectal cancer.

In this work, we studied *ezrin* expression by immunohistochemical staining of 51 cases of colorectal carcinoma and we found that it was higher in CRC tissues than that in the adjacent normal colorectal mucosa. Similar results were obtained by Toms et al., (2012); Wang et al., (2012).

In our study, *ezrin* was expressed in 92.2% of the studied cases of colorectal carcinoma. There was a statistically significant relation between *ezrin* expression and tumor grade (p-value <0.05). Similar result was obtained by Wang et al., (2009) and Patara et al., (2011). Against this finding, Lin and Chen, (2013) and Fathi et al., (2017) reported that no significant relationship was found between ezrin expression and degree of differentiation of colorectal carcinoma in their studied cases. This may be attributed to different sample size.

In the current study, there was no significant relation between *ezrin* expression and histopathological type, gender and tumor site. Similar results were obtained by Patara et al., (2011); Jin et al., ( 2012) and Fathi et al., (2017). 

As regard age, no statistical significant relationship was found between *ezrin* expression and age of the studied cases. These results were contrary to Lin and chen (2013) and Fathi et al., (2017).

Variation among results of different studies including the current one is probably attributed to the different study samples as regards the number of the study population, the race and socioeconomic standard of the patients.

In our study, All cases that showed negativity to ezrin was > 40 years old and all cases ≤ 40 years is positive to ezrin. This matches with the concern of CRC in which tumors affecting the younger population (<40 years old) is attached to poor prognosis. The rate of lymphatic metastasis in patients less than 40 years of age are higher due to the rapid progression of the disease in young patients (Pal, 2006).

Reports from Europe demonstrate that the 5 year survival rate for young patients (30 years old or younger) is only 25–30% (Miyaka et al., 2002). 

As regard tumor size, no statistical relationship was found between *ezrin* expression and tumor size. Same results were reported by Lin and chen (2013). In contrast to our study, Fathi et al., (2017) reported that relation between *ezrin* expression and tumor size is statistically significant. Variation among studies may be attributed to sample size.

As regard T-stage (tumor invasion), no significant relationship was found between *ezrin* expression and T-stage. In contrast to our study, Fathi et al., (2017) reported that there was a significant association between increasing the depth of invasion and the overexpression of *ezrin*. 

Conversely, another study made by Len and chen et al., (2013) revealed that *ezrin* expression is inversely proportion to depth of invasion. They reported that *ezrin* expression in cases without serosal invasion were significantly higher than CRC cases with serosal invasion. 

In the current study, no significant relationship was found between *ezrin* expression and L.N metastasis. In contrast to our study, Wang et al., (2009), Lin and chen (2013) and Fathi et al., (2017) reported that there was a significant relationship between *ezrin *expression and L.N metastasis. 

As regard distant metastasis, *ezrin* expression was positive in the only studied case with distant metastasis (M1) and its expression was strong. *Ezrin* expression was positive in 92% of cases without distant metastasis (M0). Our study showed no statistical relation between *ezrin* expression and distant metastasis. This was in contrary to what reported by Wang et al., (2009) and Fathi et al., (2017).

In this work we scanned the positively charged ezrin immunostained slides by Biolomagene slide scanner and snapshots were selected from the obtained virtual slides and underwent digital quantitative analysis of ezrin immunostain. A comparative study was done between the quantitative immunohistochemical staining measured by digital image and our visual scoring results (objective analysis).

In our study* ezrin* expression using objective method of analysis was strongly positive in 56.9%, weakly positive in 35.3% and negative in 7.8% of the studied cases. However its expression using the quantitative method was strongly positive in 39.2%, weakly positive in 54.9% and negative in 5.9% of the studied case.

Comparison between *ezrin* expression using quantitative method and objective method revealed that 3 cases were negative when using both methods while one case was negative when analyzed by objective method and was weakly positive when analyzed by quantitative method of analysis. This variation may be attributed to the fact that the low level staining that is present and quantifiable by digital methods may be interpreted as “negative” by a pathologist relying on visual interpretation of staining intensity resulting in misclassification (McCabe et al., 2005). 

These results ensures one of the advantages of digital scoring (quantitative method) over visual scoring (objective method) as reported by Bloom and Harrington (2004); Rimm et al., (2007) and Gavrielides et al., (2011) who stated that digital methods overcome many of the limitations of visual scoring as it allows algorithmic parameters to be locked yields more reproducible data especially when the staining is most linearly related to antigen concentration. 

In the current study 17 cases were weakly positive when analyzed by both methods while one case gave weakly positive score when analyzed by objective method and strongly positive score with quantitative method.

This emphasis that digital pathology is better than visual pathology which is fraught with problems due to subjectivity in interpretation. Digital scanning promise to overcome these limitations as the glass slide is converted into diagnostic quality digital images (Yagi and Gibertson, 2008) and the automated IHC measurements are precise in ranges of staining that appear weak to the eye (Rizzard et al., 2012).

In this work 19 cases were strongly positive when analyzed by both methods while 10 cases showed strongly positive expression when using objective method and weakly positive expression when using quantitative method. This difference was explained by Rimm et al., (2007) who reported that the human eye is least accurate at detecting differences under conditions of weak staining at which IHC is most linearly related to target antigen concentration. Consequently, regions of negative and high-positive intensities may be overcalled leading to artificially-produced bimodal score distributions.

Our study revealed that there was a statistically significant relation between ezrin objective analysis score and ezrin quantitative analysis score (P-value <0.05). A strong positive Pearson correlation exists between both methods of analysis (R=0.868).

Similar results were obtained by numerous studies that have demonstrated a high degree of correlation between digital image analysis and pathologist visual scoring. The majority of this research has been performed in breast cancer tissue on estrogen receptor, progesterone receptor (Turbin et al., 2008; Faratian et al., 2009; Bolton et al., 2010; Krecsak et al., 2011), human epidermal growth factor receptor (Atkinson et al., 2011; Ayad et al., 2015), Ki 67 assessment in breast cancer (Ayad et al., 2018).

Similar strong correlations between digital image score (quantitative method) and pathologist visual scoring (objective method) have been described in different tissue types other than breast including epidermal growth factor receptor signaling molecule in colorectal cancer (Messersmith et al., 2005), cell-free DNA level in ovarian cancer (Rizzardi et al., 2012), DNA mismatch repair protein in esophageal cancer (Alexander et al., 2012) and prognostic value of changes in quality life scores in prostate cancer (Braun et al., 2013).

The complete digitalization of a slide has the potential to transform the practice of diagnostic pathology.

The statistical analysis revealed significant relation between *ezrin* expression and tumor grade. This points to the role of ezrin in colorectal cancer progression and that ezrin might provide clinically valuable information in predicting the behavior of colorectal cancer. A statistically significant relation between ezrin objective analysis score and ezrin quantitative analysis score (P-value <0.05) and a strong positive Pearson correlation existed between both methods of analysis (R=0.868). This concludes that digital pathology offers the potential for improvements in quality, efficacy and safety that are compelling reasons for widespread implementation. 

**Table 1:** Relation between Immunohistochemical Expression of Ezrin and Different Clinicopathological Parameters when Analyzed by Objective Method of Analysis

			Ezrin expression (Objective analysis)			P value
			Negative	Weakly positive	Strongly positive	
		Total (n/%)	N (%)	N (%)	N (%)	
Gender	Male	[(25 (49)]	2 (8)	10 (40)	13 (52)	0.774
	Female	[(26 (51)]	2 (7.7)	8 (30.8)	16 (61.5)	
Tumor Grade	Grade II	[(44 (86.3)]	4 (9.1)	18 (40.9)	22 (50)	0.046
	Grade III	[(7 (13.7)]	0 (0)	0 (0)	7 (100)	
Tumor stage	Stage 2	[(7 (13.7)]	1 (14.3)	5 (71.4)	1 (14.3)	0.154
	Stage 3	[(37 (72.5)]	2 (5.4)	11 (29.7)	24 (64.9)	
	Stage 4	[(7 (13.7)]	1 (14.3)	2 (28.6)	4 (57.1)	
Lymph node Stage	Negative	[(26 (51)]	3 (11.5)	10 (38.5)	13 (50)	0.292
	Stage 1	[(9 (17.6)]	0 (0)	5 (55.6)	4 (44.4)	
	Stage 2	[(16 (31.4)]	1 (6.3)	3 (18.8)	12 (75)	
Distant Metastasis	Negative	[(50 (98)]	4 (8)	18 (36)	28 (56)	0.679
	Positive	[(1 (2)]	0 (0)	0 (0)	1 (100)	
Lymph node Metastasis	Negative	[26 (51)]	3 (11.5)	10 (38.5)	13 (50)	0.469
	Positive	[(25 (49)]	1 (4)	8 (32)	16 (64)	
Tumor Site	Left side	[(11 (21.6)]	2 (18.2)	5 (45.5)	4 (36.4)	0.107
	Rectum	[(5 (9.8)]	0 (0)	2 (40)	3 (60)	
	Right side	[(21 (41.2)]	0 (0)	4 (19)	17 (81)	
	Sigmoid	[(10 (19.6)]	1 (10)	6 (60)	3 (30)	
	Transverse colon	[(4 (7.8)]	1 (25)	1 (25)	2 (50)	
Gross appearance	Fungating	[(20 (39.2)]	1 (5)	9 (45)	10 (50)	0.418
	Infiltrating	[(10 (19.6)]	1 (10)	1 (10)	8 (80)	
	Ulcer	[(21 (41.2)]	2 (9)	8 (38.1)	11 (52.4)	
Microscopic diagnosis	Adenocarcinoma	[(45 (88.2)]	4 (8.9)	17 (37.8)	24 (53.3)	0.735
	Mucinous	[(3 (5.9)]	0 (0)	0 (0)	3 (100)	
	neuroendocrine	[(1 (2)]	0 (0)	0 (0)	1 (100)	
	Papillary adenocarcinoma	[(2 (3.9)]	0 (0)	1 (50)	1 (50)	
Age category	Age category	[(5 (9.8)]	0 (0)	1 (20)	4 (80)	0.516
	> 40 years	[(46 (90.2]	4 (8.7)	17 (37)	25 (54.3)	
Tumor size category	≤ 5	[(28 (54.9)]	2 (7.1)	12 (42.9)	14 (50)	0.459
	> 5	[(23 (45.1)]	2 (8.7)	6 (26)	15 (65.2)	

**Table 2:** Relation between Immunohistochemical Expression of Ezrin when Analyzed by Objective Method of Analysis and when Analyzed by Digital Quantitative Assessment

		Ezrin expression (Objective analysis)			*P*-value
		Negative	Weakly positive	Strongly positive	
		N (%)	N (%)	N (%)	
Ezrin expression	Negative	3 (100)	0 (0)	0 (0)	<0.001
(Quantitative analysis)	Weakly positive	1 (3.6)	17 (60.7)	10 (35.7)	
	Strongly positive	0 (0)	1 (5)	19 (95)	

**Figure 3:**
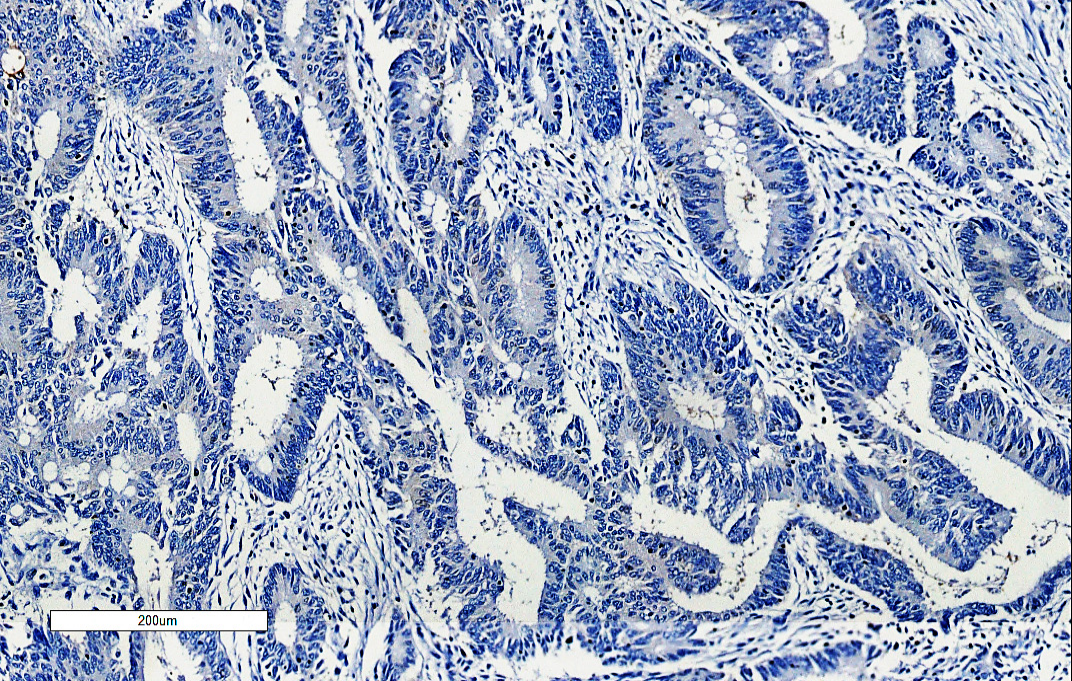
Moderately Differentiated Adenocarcinoma Showing Negative Ezrin Staining Intensity (IHC Staining x 100).

**Figure 4:**
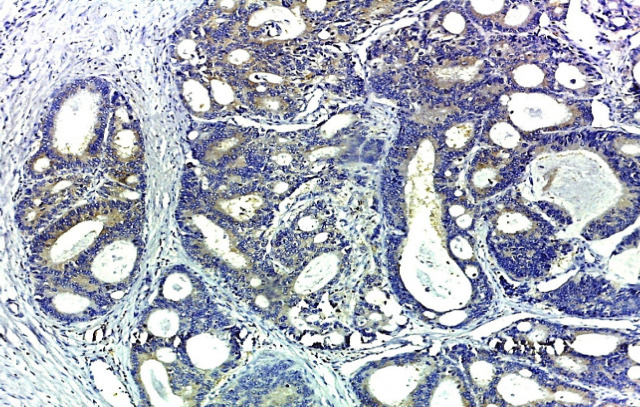
Moderately Differentiated Adenocarcinoma Showing Weak Cytoplasmic Ezrin Staining Intensity (IHC Staining x 200).

**Figure 5:**
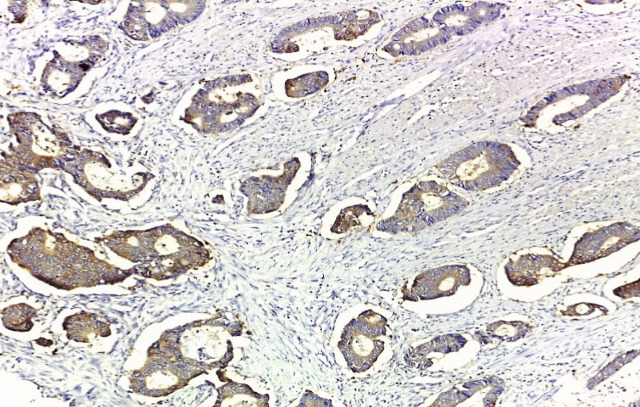
Moderately Differentiated Adenocarcinoma Showing Moderate Cytoplasmic Ezrin Staining Intensity (IHC Staining x 200).

**Figure 6:**
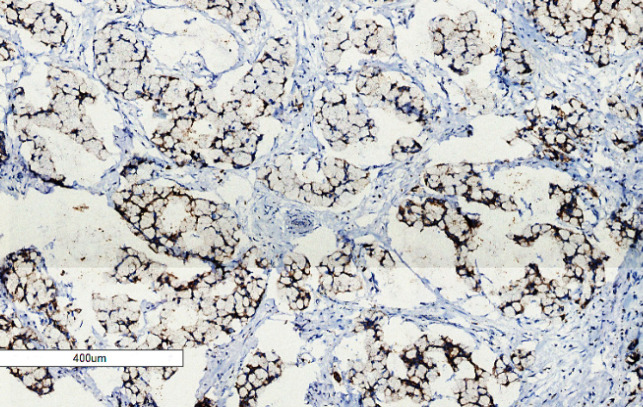
Mucinous Adenocarcinoma with Signet Ring Features Showing Strong Cytoplasmic Ezrin Staining Intensity (IHC Staining x 50).
